# Annual Plasma Neurofilament Dynamics Is a Sensitive Biomarker of Disease Activity in Patients with Multiple Sclerosis

**DOI:** 10.3390/medicina59050865

**Published:** 2023-04-29

**Authors:** Miriam Fedičová, Pavol Mikula, Zuzana Gdovinová, Marianna Vitková, Norbert Žilka, Jozef Hanes, Lýdia Frigová, Jarmila Szilasiová

**Affiliations:** 1 Department of Neurology, Faculty of Medicine, Pavol Jozef Šafárik University, 040 11 Košice, Slovakia; 2 Department of Social and Behavioural Medicine, Faculty of Medicine, Pavol Jozef Šafárik University, 040 11 Košice, Slovakia; 3 Institute of Neuroimmunology, Slovak Academy of Science, 845 10 Bratislava, Slovakia; 4 Magnetic Resonance Imaging, ProMagnet, 041 91 Košice, Slovakia

**Keywords:** multiple sclerosis, disease activity, neurofilament light chain, neurofilament dynamics, NEDA

## Abstract

*Background and Objectives*: Neurofilament light chain (NfL) is a sensitive biomarker of neuroaxonal damage. This study aimed to assess the relationship between the annual change in plasma NfL (pNfL) and disease activity in the past year, as defined by the concept no evidence of disease activity (NEDA) in a cohort of multiple sclerosis (MS) patients. *Materials and Methods:* Levels of pNfL (SIMOA) were examined in 141 MS patients and analyzed in relationship to the NEDA-3 status (absence of relapse, disability worsening, and MRI activity) and NEDA-4 (NEDA-3 extended by brain volume loss ≤ 0.4%) during the last 12 months. Patients were divided into two groups: annual pNfL change with an increase of less than 10% (group 1), and pNfL increases of more than 10% (group 2). *Results:* The mean age of the study participants (*n* = 141, 61% females) was 42.33 years (SD, 10.17), and the median disability score was 4.0 (3.5–5.0). The ROC analysis showed that a pNfL annual change ≥ 10% correlates with the absence of the NEDA-3 status (*p* < 0.001; AUC: 0.92), and the absence of the NEDA-4 status (*p* < 0.001; AUC: 0.839). *Conclusions:* Annual plasma NfL increases of more than 10% appear to be a useful tool for assessing disease activity in treated MS patients.

## 1. Introduction

Multiple sclerosis (MS) is an autoimmune disease with variable activity and response to therapy. Many studies in recent years have analyzed the sensitivity of blood biomarkers in relation to current and future disease activity [1,2,3,4,5]. The plasma or serum level of the neurofilament light chain (NfL) is a biomarker of neuroaxonal damage in multiple sclerosis [1,4,5,6,7]. Significant associations between NfL concentrations and disease activity determined by NEDA status (no evidence of disease activity) have been shown in several studies [2,3,5,8,9,10]. Fulfilling the criteria of the NEDA-3 status is considered in clinical practice to be the optimal effectiveness of disease modifying therapy (DMT) [11]. The NEDA-3 status means the absence of relapse, brain magnetic resonance imaging (MRI) activity, and disability worsening during the last 12 months. NEDA-4 is NEDA-3 extended by annual brain volume loss ≤ 0.4%. However, neither the NEDA-3 status nor NEDA-4 may cover ongoing neurodegenerative processes, overlooking patients with a progression independent of relapse activity (PIRA) [12]. An individual assessment of the annual difference in NfL concentrations, i.e., the NfL dynamics, appears to be a possible solution to this limitation. NfL dynamics have been used in clinical drug studies as an outcome parameter and have shown that the effect of drugs on NfL dynamics was associated with clinical and MRI outcomes [3]. A decrease in NfL levels is an important indicator of MS treatment efficacy. Studied cohorts of patients with MS and NfL evaluation have mostly been focused on the early disease stage [6,13,14,15,16,17]. In our previous work, we found that a cut-off value of 10.1 pg/mL for pNfL (plasma NfL) might differentiate patients with and without the activity of disease expressed by NEDA-3 [18,19]. In this study, we attempted to clarify whether the annual change of pNfL (an increase of 10% and more) is a sensitive biomarker related to disease activity in the last 12 months, as defined by the NEDA status. Plasma NfL dynamics were assessed in relation to demographic, clinical, and radiological outcomes, including NEDA-3 and NEDA-4 status in our MS cohort. To the best of our knowledge, the mentioned relationships have not been more extensively investigated in previous studies.

## 2. Materials and Methods

### 2.1. Patient Recruitment

**Figure 1:**
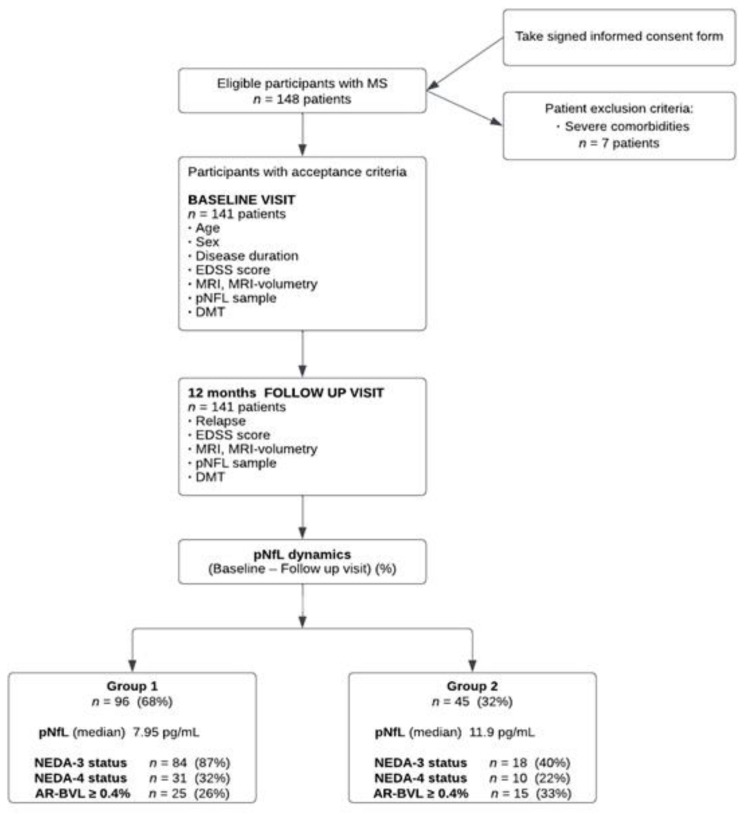
Study flow diagram shows the procedures applied to MS patients and their distributions into groups. MS, multiple sclerosis; EDSS, expanded disability status scale; MRI, magnetic resonance imaging; pNfL, plasma neurofilament light chain; DMT, disease-modifying therapy; NEDA, no evidence of disease activity; BVL, brain volume loss.

This observational prospective study was conducted in the Department of Neurology of Pavol Jozef Šafárik University Medical Faculty and Louis Pasteur University Hospital in Košice, from January 2019 to June 2022. A total of 148 consecutive patients with MS were recruited in this study and 141 fulfilled the inclusion criteria (Figure 1).

The inclusion criteria were: (1) the diagnosis of relapsing multiple sclerosis based on the McDonald criteria (2010); (2) age over 18 years; (3) agree to enter the study and provide written informed consent; and (4) no relapse of MS at least two months prior to the baseline visit. Severe comorbidity affecting the nervous system which may have led to an increased NfL level was the exclusion criterion. Seven patients were therefore excluded from the study: four patients with stroke in the past year, one patient with neurofibromatosis type I, one patient with spinal muscular atrophy, two patients with diabetes mellitus, and one patient with small-vessel disease. Throughout the study, all patients were treated with DMT according to the national MS treatment guidelines.

### 2.2. Study Methods

Each patient was examined in two visits (a baseline visit and a follow-up visit—FUV) over a distance of 12 months. The visit involved a clinical examination with expanded disability status scale (EDSS) assessment [20], plasma NfL sampling, and brain MRI. Disease duration was the time from the first occurrence of MS symptoms to the date of the baseline visit. Each patient enrolled in the study was instructed to immediately contact the investigator in case of any worsening within the research period. The relapse of MS was treated with corticosteroids according to standard procedures, and the patient was checked 3 months and 6 months after relapse. Once the sustained disability worsening was confirmed after 6 months, it was recorded in the FUV.

#### 2.2.1. NfL Measurements

Samples for NfL assessment were processed as follows: 3 milliliters of blood were collected and left at room temperature within 120 min, then centrifuged at 4000 rpm for 10 min. Then, the collected plasma samples were stored in a polypropylene tube at a temperature of −80 °C. pNfL concentrations were analyzed using SIMOA^TM^ (Single Molecule Array, NF-Light Advantage Kit, SIMOA HD-1 analyzer, protocol Quanterix, Lexington, MA, USA) [18,19,21]. For each patient, pNfL was measured in two plasma samples, at the baseline visit, and at the FUV after 12 months. The pNfL dynamics were calculated as their arithmetic difference expressed as a percentage. Patients were divided into two groups: group 1 contained patients who had either any annual decrease in pNfL levels or an annual increase in pNfL levels up to 10%. Group 2 included patients with an annual pNfL increase of more than 10%. Within group 2, patients with an annual pNfL increase of more than 20% were categorized as subgroup 2A.

#### 2.2.2. MRI Measurements

All patients underwent brain MRI on the same device, namely PHILIPS Ingenia 3.0T Omega HP (Philips Medical Systems, Best, The Netherlands). The MRI protocol involved sequences as follows: 3D T1-weighted magnetization-prepared rapid gradient-echo, and a 3D T2-weighted FLAIR sequence. Longitudinal co-registration fusion was used for the detection of new or enlarged T2-weighted, FLAIR, Gd-enhancing, and T1-weighted lesions. Volumetric parameters were calculated by the automatic volume quantification of FLAIR and T1-weighted sequences in the program Icobrain (Leuven, Belgium) [22]. We evaluated the following brain volumetric parameters: whole brain (WB) volume, grey matter (GM) volume, annual brain volume loss (BVL), volume of FLAIR lesions, T2-weighted lesions, T1-weighted lesions, T2- and T1-weighted lesions annual volume change, and normative percentile of the WB volume and GM volume. The threshold for the annual brain volume loss rate was 0.4%, according to the definition by De Stefano et al. [23]. MRI measures were performed at baseline and follow-up visit within the range of 1–3 months. MRI scans were evaluated by blinded radiologists.

#### 2.2.3. NEDA Definition

Disease activity and NEDA status were evaluated at the FUV. NEDA-3 means that the patient had no relapse, disability (EDSS score) worsening, or MRI activity for the last 12 months. Disability worsening was defined as an increase in the EDSS score of 1.5 points if the baseline EDSS score was 0; an increase of ≥1.0 point if the baseline EDSS score was ≤5.0, or an increase of ≥0.5 points if the baseline EDSS was ≥5.5. This increase in EDSS score had to persist for at least 6 months [24]. The MRI activity was defined as the presence of at least 2 or more new/or enlarged lesions in a T2-weighted scan or the presence of a Gd-enhancing T1-lesion on the FUV MRI scan compared with the baseline MRI scan. NEDA-4 status means that the patient had the presence of a NEDA-3 status, and at the same time, had an AR-BVL ˂ 0.4% in brain volumetry [23].

### 2.3. Data Analysis

Demographic, clinical, NfL, and MRI data of patients were calculated as a number (percentage), mean (standard deviation), and median (CI). Pearson’s correlation coefficient was used to calculate the relationships between variables under study. The receiver operating characteristics (ROC) curve analysis was used for the assessment of annual pNfL dynamics as a predictor of the status NEDA-3 and NEDA-4. The area under the curve (AUC) with a 95% CI was calculated. From the ROC curve analysis, we found a cut-off value for the pNfL dynamics (10 %), which has the potential to differentiate patients with and without NEDA-3 status. Based on the cut-off value of 10%, the total cohort of patients was divided into two groups, 1 and 2. Next, a comparison between groups 1 and group 2 was performed using a *t*-test. Differences were considered significant at *p* ˂ 0.05. Data analyses were performed using IBM SPSS (software version 26.0).

### 2.4. Ethical Considerations

All patients signed an informed consent form and agreed to the use of their anonymized data for research. The Ethics Committee of L. Pasteur University Hospital in Košice approved this study on 19 April 2017 (no. 2017/EK/4005).

## 3. Results

The demographic, clinical, MRI, and laboratory characteristics of the study sample are presented in Table 1.

All patients were treated with DMTs: 34 (24%) were treated with first-line DMTs and 107 (76%) with second-line DMTs. Patients were treated as follows: 5 (3.4%) with teriflunomide, 4 (2.7%) with interferon-beta, 1 (0.6%) with glatiramer acetate, 24 (17%) with dimethyl fumarate, 20 (14%) with natalizumab, 19 (13.4%) with fingolimod, 21 (14.9%) with ocrelizumab, 36 (25.5%) with cladribine, and 12 (8.5%) with alemtuzumab.

**Figure 2:**
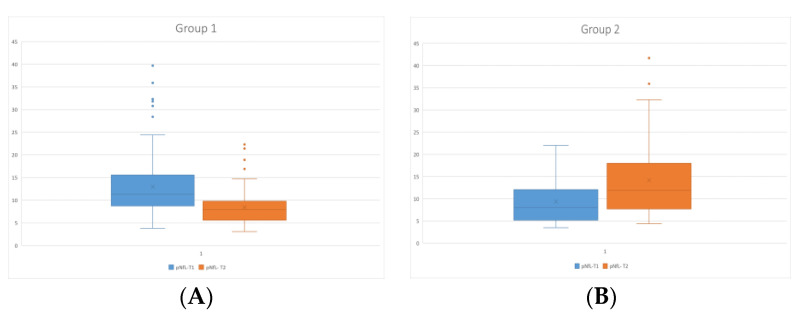
Comparison of the pNfL levels at the baseline visit (pNfL-T1) and follow-up visit (pNfL-T2) in group 1 (**A**) and in group 2 (**B**). pNfL—plasma neurofilament light chain; T1—baseline; T2—follow-up.

The comparison of the pNfL levels at the baseline visit (NfL-T1) and FU visit (NfL-T2) between groups 1 and 2 showed significant differences, with the decrease in group 1 (mean pNfL-T1 = 13.02 ± 6.99 pg/mL vs. mean pNfL-T2 = 8.48 ± 3.8 pg/mL, *p* ˂ 0.001) and with the increase in group 2 (mean pNfL-T1 = 9.36 ± 4.89 pg/mL vs. mean pNfL-T2 = 14.15 ± 8.36 pg/mL, *p* ˂ 0.001) (Figure 2A,B).

**Figure 3:**
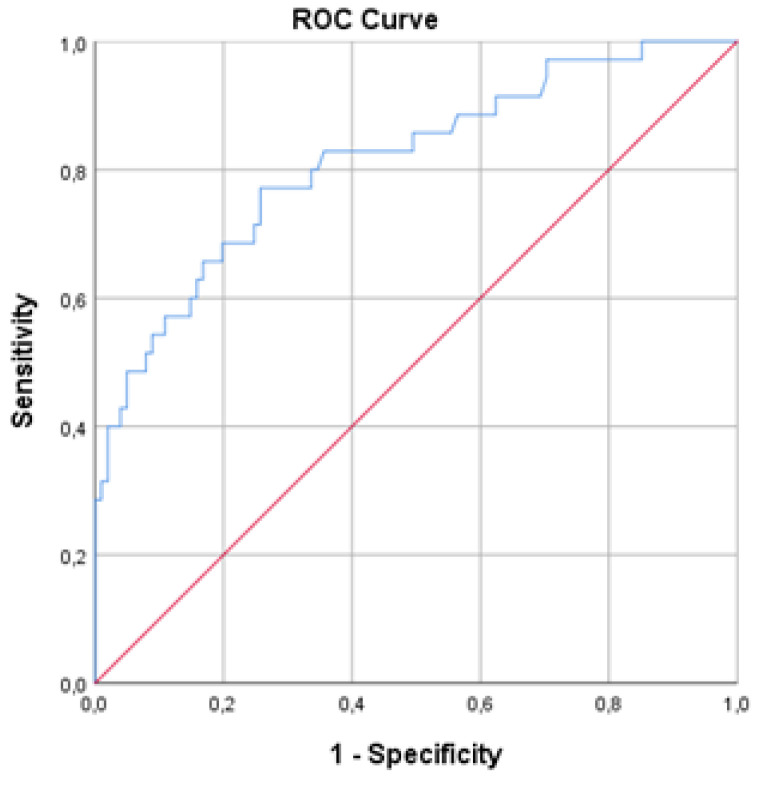
ROC curve of the pNfL dynamics and NEDA-3 status.

ROC curve analysis showed that, among all the patients in the cohort, the pNfL dynamics variable correlates with NEDA-3 status (AUC = 0.813; 95% CI = 0.726–0.9; *p* < 0.001; the sensitivity and specificity were 77% and 74%, respectively). Lower pNfL dynamics were associated with a higher probability of achieving NEDA-3 status, and a cut-off level for pNfL dynamics was 11% (Figure 3).

Ninety-six (68%) patients either had an annual decrease in pNfL levels or an annual increase in pNfL levels up to 10% (group 1), and 45 (32%) patients had an annual increase in pNfL above 10% (group 2). The characteristics of the entire study group for groups 1 and 2 are provided in Table 1.

A significant correlation was found between pNfL dynamics and EDSS score at FUV (r = 0.34, *p* ˂ 0.001) and annual volume change of WB (r = −0.36, *p* ˂ 0.01), as well as between pNfL at FUV and age (r = 0.39, *p* ˂ 0.001), disease duration (r = 0.19, *p* ˂ 0.05), EDSS score at FUV (r = 0.42, *p* ˂ 0.001), time from the last relapse (r = −0.32, *p* ˂ 0.01), annual volume change of WB (r = −0.43, *p* ˂ 0.001), volume of FLAIR-lesions (r = 0.35, *p* ˂ 0.01) and volume of T1-lesions (r = 0.32, *p* ˂ 0.001). From a total of 141 patients, 102 (72%) achieved NEDA-3 status, and 41 (29%) patients achieved NEDA-4 status (Figure 1). We found that 38 (84%) patients from group 2 had an increase in pNfL above 20% (subgroup 2A), and 11 (29%) of them achieved NEDA-3 status and 8 (21%) patients achieved NEDA-4 status.

Patients in group 2 had significantly higher pNfL levels in comparison to group 1 (*p* < 0.001), pNfL dynamics (*p* < 0.001), annual change of WB volume (*p* < 0.001), annual change of GM volume (*p* < 0.001), significantly lower number of patients with NEDA-3 (*p* < 0.001) and NEDA-4 status (*p* < 0.05), higher proportion of patients with last year relapse (*p* < 0.001), higher number of patients with EDSS worsening (*p* < 0.001), and higher number of patients with MRI activity last year (*p* < 0.01) (Figure 1, Table 1).

**Figure 4:**
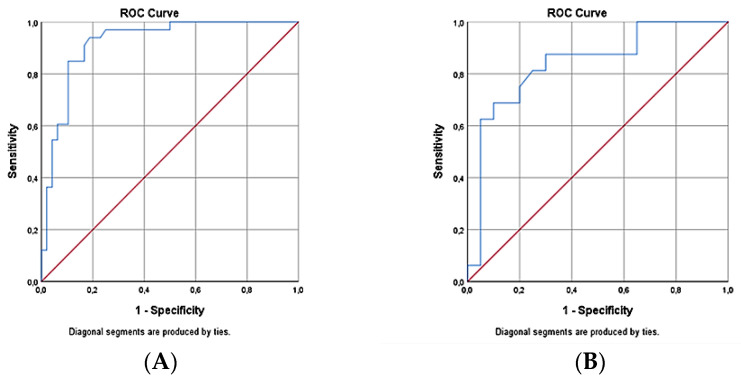
ROC curve of the pNfL dynamics and NEDA-3 status in group 2 (**A**): ROC curve of the pNfL dynamics and NEDA-4 status in group 2 (**B**).

The ROC curve analysis shows in group 2 (pNfL increase ≥ 10%) that the pNfL dynamics correlate with the NEDA-3 status (AUC = 0.92; 95% CI = 0.86–0.98; *p* < 0.001; the sensitivity and specificity were 84.8% and 85.4%, respectively). Lower values of pNfL dynamics were associated with a higher probability of achieving the NEDA-3 status, and the cut-off for pNfL dynamics was 10% (Figure 4A). The ROC curve analysis showed in group 2 that the pNfL dynamics correlate with NEDA-4 status (AUC = 0.839; 95% CI = 0.702–0.976; *p* < 0.001; the sensitivity and specificity were 81.3% and 75%, respectively). Lower values of pNfL dynamics were associated with a higher probability of achieving the NEDA-4 status, and the cut-off for pNfL dynamics was 9.9% (Figure 4B).

**Figure 5:**
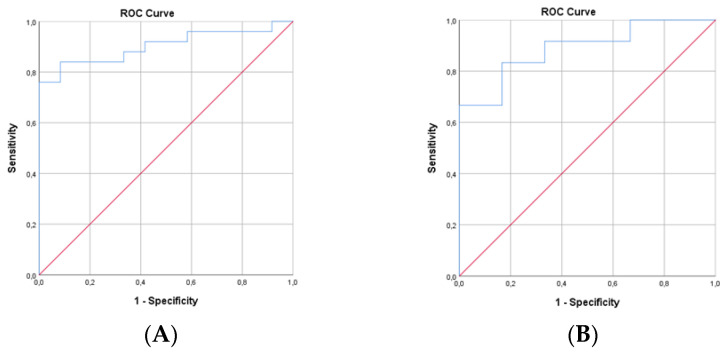
ROC curve of the pNfL dynamics and NEDA-3 status in subgroup 2A (**A**). ROC curve of the pNfL dynamics and NEDA-4 status in subgroup 2A (**B**).

The ROC curve analysis shows in subgroup 2A (pNfL increase ≥ 20%) that the pNfL dynamics correlate with the NEDA-3 status (AUC = 0.903; 95% CI = 0.806–1.0; *p* < 0.001; the sensitivity and specificity were 88% and 67%, respectively). Lower values of pNfL dynamics were associated with a higher probability of achieving NEDA-3 status, and a cut-off for pNfL dynamics was 39.4% (Figure 5A). The ROC curve analysis shows in subgroup 2A (pNfL increase ≥ 20%) that the pNfL dynamics correlate with the NEDA-4 status (AUC = 0.889; 95% CI = 0.736–1.0; *p* < 0.01; the sensitivity and specificity were 83% and 83.3%, respectively). The lower values of pNfL dynamics were associated with a higher probability of achieving the NEDA-3 status, and a cut-off for pNfL dynamics was 39.4% (Figure 5B).

## 4. Discussion

The results of this study show that the annual change in pNfL level could be a sensitive indicator of disease activity present in the past 12 months in a patient with MS, assessed using the NEDA-3 concept, which requires the absence of clinical relapse, disability worsening, brain MRI activity, and the NEDA-4 concept (NEDA-3 extended by the annual rate of BVL ≤ 0.4%). The most important finding of our study is the association between NEDA-3 status, also known as the “gold standard” evaluation of multiple sclerosis disease activity, and the annual dynamics of pNfL, as determined by the analysis of the area under the ROC curve (AUC = 0.813; *p* < 0.001). Taking into account the findings of some authors that the annual increase in NfL in healthy persons varies from 2.2% up to 3.3% [6], as well as the results of our ROC curve analysis (lower pNfL dynamics are associated with a higher probability of achieving NEDA-3 status, with a cut-off level of 11% for pNfL dynamics), we chose a range of 10% for a pathological annual increase.

While individual levels of pNfL at FUV correlated with variables such as patient age, the duration of the disease, EDSS score, time from the last relapse from baseline to FUV, the whole brain annual volume change, FLAIR-lesions volume, and T1-lesions volume, a significant correlation was found between annual pNfL dynamics and EDSS at FUV and the whole-brain annual volume change at FUV. Not surprisingly, in group 1 (either any annual decrease or an annual increase in pNfL levels of up to 10%) was achieved NEDA-3 status in 87% and NEDA-4 status in 32% of patients. We could consider these patients as being effectively treated with DMT.

An important finding supporting pNfL as a biomarker of MS activity was the group 2 analysis showing that patients with an annual increase in pNfL of more than 10% had significantly lower proportions of NEDA-3 (40% vs. 87%) and NEDA-4 (22% vs. 32%) and a significantly higher proportion of relapse in the last year (40% vs. 8%) and EDSS worsening (42% vs. 6%) in comparison with group 1. The occurrence of accelerated annual brain atrophy (AR-BVL ≥ 0.4%) was different in groups 1 and 2 (33% vs. 26%). One of the possible explanations for this finding is that an apparent decrease in brain volume appears with a certain delay after inflammatory events such as new or enlarged brain lesions in MRI and clinical relapse. Our results show that, despite a pathological increase in pNfL, up to 40% of patients in group 2 achieved a NEDA-3 status, and 22% met the criteria for NEDA-4 status. By excluding the presence of comorbidity that would explain this pNfL increase, these results suggest that pNfL dynamics might be related to the so-called hidden “silent progression”, and these cases may include patients with the progression that is not associated with relapse or MRI activity.

We have to admit the possibility of insufficiently capturing the beginning of disability worsening (cases with a possible “false positive” NEDA-3 status). This is a known shortcoming of the widely used EDSS scale because it does not sufficiently cover all attributes of disability [8,25,26]. Disability worsening in those patients who met the criteria of NEDA-3 status could be caused by brain and spinal cord atrophy [6]. Cross-sectional studies with static measures of NfL levels have shown associations between higher NfL and disability progression, the onset of a secondary progressive MS, MRI activity, and accelerated brain atrophy [3,6,27,28]. Several studies aimed to monitor MS activity presenting a high inter-individual distribution of NfL levels and recommended longitudinal measurements of intra-individual NfL changes as a more appropriate approach for assessing disease activity [5,6,13,29,30].

For clinical practice, it is necessary to answer “when” to measure NfL and “how should the findings be integrated into clinical decision-making?” Only a few studies to date have focused on detecting the NfL threshold rate associated with a clinically significant change in longitudinal NfL measures [5,6]; however, they mainly focused on establishing cut-offs for absolute NfL values and did not evaluate differences in NfL levels in percentages. Therefore, we decided to determine the threshold values (% of NfL dynamics) that would detect disease activity in the last 12 months. Our ROC analysis showed that the increase in pNfL is associated with the absence of NEDA-3 and NEDA-4 status, with a cut-off of 10% for pNfL dynamics. The ROC analysis in subgroup 2A showed that pNfL dynamics with an increase of over 20% is predictive of the absence of NEDA-3 status and NEDA-4 status, with a higher sensitivity in the predictive model than was shown in a model with an annual change above 10% (sensitivity of 88% vs. 84% for NEDA-3; sensitivity of 83% vs. 81% for NEDA-4). We conclude that the pNfL dynamics with an increase of 20% or more appear to be a slightly more sensitive indicator of disease activity in the last 12 months. In previous work, we found that a cut-off value of 10.1 pg/mL for pNfL allows to discriminate between patients with and without NEDA-3 status [18]. Taking into account the fact that individual pNfL values can be permanently higher in some patients, pNfL dynamics assessment therefore appears to be a better biomarker of disease activity.

NEDA-3 status is considered a criterion for effective treatment in patients with MS and is an important factor in decisions regarding therapy continuation or switching. The main disadvantage is that the NEDA-3 concept mainly covers the inflammatory component of MS, and little, if any, covers the neurodegenerative process [25]. Our results show that the pNfL dynamics with an increase above 10% constitute a sensitive biomarker of disease activity in patients with MS. A significant annual increase in pNFL levels could help detect patients with progression independent of relapse activity (PIRA). This is important information that may help in making a decision on therapeutic strategies. In contrast with other studies with cohorts of early MS, our study sample consists of patients with ongoing disease and DMT, with a relatively older age, and a relatively long disease course.

We assume that annual NfL dynamics along with clinical and radiological tools might be a sensitive biomarker allowing us to discriminate between those patients with no disease activity and patients with disease activity in last 12 months. Our results support suggestions that the serial NfL assessment could be an alternative to annual brain MRI scans in clinically stable patients.

### Limitations

This study has several limitations. Our cohort was heterogenous from a DMT point of view, and as groups 1 and 2 were small, our results only allow us to draw conclusions with caution. We need larger and more homogenous cohorts for a longitudinal serial NfL samples collection for a better explanation of pNfL validity in the individual MS course. We are aware that all comorbidities, as well as aging, body weight, and cardiovascular risk factors, can affect the measured NfL levels [31]. An annual increase in pNfL dynamics of only 10% does not have absolute significance. In patients with very low baseline pNfL values (e.g., lower than 10 pg/mL), it is unlikely that a 10% annual increase would reflect a clinically significant change.

## 5. Conclusions

In the present work, we demonstrated that the plasma neurofilament annual increase above 10% is a sensitive biomarker related to disease activity in treated patients with relapsing MS. The annual increase in pNfL of more than 10% is associated with clinical and MRI activity, including brain volumetry in the last 12 months. Plasma NfL might be a promising blood biomarker added to personalized medical approaches in patients with MS.

## Figures and Tables

**Table 1:** Characteristics of the study sample.

Variable	Total	Group 1	Group 2	Difference between Groups 1 and 2 (*p* Value)
NfL dynamics		Decrease or increase ˂10%	Increase ≥10%	Sig.
Demographic characteristics				
Sample size, *n* (%)	141 (100)	96 (68)	45 (32)	
Age (years) at baseline visit, mean (SD)	42.33 (10.2)	42.78 (9.9)	41.36 (10.8)	
2. Clinical and laboratory characteristics				
Disease duration (years) at baseline visit, median, IQR	12 (8.5–18)	12 (7.3–18)	12 (9–18.5)	
EDSS score at FUV, median, IQR	4.0 (3.5–5)	4 (3–5)	4.3 (3.5–5)	
pNfL at FUV, pg/mL, median, IQR	8.9 (6.3–11.9)	7.95 (5.6–9.8)	11.9 (7.7–18)	*p* < 0.001
pNfL dynamics at FUV, %, median, IQR	−10.3 (−37.4–25)	−25 (−46.0–−9.1)	44.4 (26.6–83.4)	*p* < 0.001
Patients with relapse last 12 months at FUV, *n* (%)	26 (18.4)	8 (8.3)	18 (40)	*p* < 0.001
Patients with EDSS worsening at FUV, *n* (%)	25 (17.7)	6 (6.3)	19 (42.2)	*p* < 0.001
Time from the last relapse to FUV, months, median, IQR	12 (7–21)	13 (8–20)	9 (4–24.8)	
3. MRI measures				
FLAIR-lesions volume (mL) at FUV, median, IQR	8.7 (3.8–16.7)	7.76 (3.1–15.6)	10.6 (5.9–20.3)	
FLAIR-lesions volume change (mL) at FUV, median, IQR	0.14 (−0.1– 0.5)	0.13 (−0.1– 0.42)	0.23 (0.01–0.9)	
T1-lesions volume (mL) at FUV, median, IQR	6.19 (2.3– 13.4)	5.1 (1.8–12.2)	7.04 (3.9–14.3)	
T1-lesions volume change (mL) at FUV, median, IQR	0.13 (−0.1– 0.8)	0.15 (−0.05–0.7)	0.1 (−0.03–1.1)	
WB volume (mL) at FUV, median, IQR	1494 (1439–1543)	1495 (1440–1545)	1494 (1399–1529)	
WB annual volume change (%) at FUV, median, IQR	−0.25 (−0.47–0.02)	−0.12 (−0.29–0.1)	−0.29 (−0.57– −0.02)	*p* < 0.001
WB volume normative percentile at FUV, median, IQR	5.9 (0.95–23)	6.6 (0.96–23.95)	5.05 (0.8–23.1)	
GM volume (mL) at FUV, median, IQR	902 (848–931)	902.5 (855–938)	902 (826–923)	
GM annual volume change (%) at FUV, median, IQR	−0.4 (−0.79–0.2)	−0.19 (−0.51–0.17)	−0.4 (−1.1–0)	*p* < 0.001
GM volume normative percentile at FUV, median, IQR	23.9 (6.1– 42.5)	23.5 (4.6–45.5)	24.7 (6.4–41.5)	
Patients with MRI activity last 12 months at FUV, *n* (%)	18 (12.8)	6 (6.5)	12 (26.7)	*p* < 0.01
Patients with AR-BVL ≥0.4% at FUV, *n* (%)	40 (28.4)	25 (26)	15 (33.3)	
Patients with WB atrophy at FUV, *n* (%)	26 (18.4)	52 (54)	18 (40)	
Patients with GM atrophy at FUV, *n* (%)	12 (8.5)	16 (16.7)	10 (22)	
4. NEDA				
Patients with NEDA-3 status at FUV, *n* (%)	102 (70.2)	71 (74)	18 (40)	*p* < 0.001
Patients with NEDA-4 status at FUV, *n* (%)	41 (29.1)	31 (32)	10 (22.2)	*p* < 0.05
5. DMT				
Patients with first-line DMT, *n* (%)	34 (24)	24 (25)	10 (22.2)	
Patients on second-line DMT, *n* (%)	107 (76)	72 (75)	35 (77.8)	

AR-BVL, annual rate of brain volume loss; DMT, disease modifying therapy; EDSS, expanded disability status scale; FLAIR, fluid-attenuated inversion recovery; FUV, follow-up visit; GM, grey matter; IQR, interquartile range; MRI, magnetic resonance imaging; NEDA, no evidence of disease activity; pNfL, plasma neurofilament light chain; SD, standard deviation; WB, whole brain.

## Data Availability

The data presented in this study are available upon request from the corresponding author.

## Abbreviations

MS	Multiple sclerosis
pNfL	Plasma neurofilament light chain
NEDA	No evidence of disease activity
DMT	Disease modifying therapy
MRI	Magnetic resonance imaging
PIRA	Progression independent of relapse activity
EDSS	Expanded disability status scale
BVL	Brain volume loss
RMS	Relapsing multiple sclerosis
FUV	Follow-up visit
SIMOA	Single-molecule array
FLAIR	Fluid-attenuated inversion recovery
WB	Whole brain
GM	Grey matter
AR-BVL	Annual rate of brain volume loss
Gd	Gadolinium
CI	Confidence interval
ROC	Receiver operating characteristics
AUC	Area under curve
IQR	Interquartile range
SD	Standard deviation
